# Risk factors and predictive model for intrapartum cesarean delivery in women with epidural analgesia: a retrospective cohort study

**DOI:** 10.7717/peerj.20358

**Published:** 2025-11-19

**Authors:** Yu Feng, Yili Zhao, Rui Shao, Yan Wang, Pin Zhang

**Affiliations:** 1 Department of Anesthesiology, Zhengzhou Central Hospital Affiliated to Zhengzhou University, Zhengzhou, China; 2 Legal Supervision Department, Plastic and Cosmetic Surgery Department, Zhengzhou Central Hospital Affiliated to Zhengzhou University, Zhengzhou, China; 3 Central Operating Department, Zhengzhou Central Hospital Affiliated to Zhengzhou University, Zhengzhou, China

**Keywords:** Epidural analgesia, Labor analgesia, Intrapartum cesarean delivery, Risk factors

## Abstract

**Background:**

Labor epidural analgesia (LEA) is the gold standard for labor pain management, yet its association with intrapartum cesarean delivery (CD) remains controversial. This study aimed to identify risk factors and develop a predictive model for CD conversion in parturients receiving LEA.

**Methods:**

A retrospective cohort study analyzed 1,896 parturients receiving LEA at Zhengzhou Central Hospital (2022–2023). Participants were categorized into vaginal delivery group (*n* = 1, 541) and cesarean delivery group (*n* = 355). The original dataset was randomly split into training/testing sets (7:3 ratio) for analytical purposes. Univariate analysis identified significant variables, followed by Least Absolute Shrinkage and Selection Operator (LASSO) regression and multivariate logistic regression to construct a predictive nomogram. Model performance was evaluated by area under the receiver operating characteristic curve (AUC), calibration curves, and decision curve analysis (DCA).

**Results:**

The CD conversion rate was 18.7%. Eight independent predictors were identified: height, pre-pregnancy body mass index (BMI), gestational age ≥ 40 weeks, hypertension, meperidine use, cervical dilation at analgesia initiation, intrapartum fever (≥ 38.0 °C), and estimated fetal weight. The nomogram demonstrated good discrimination (training set area under the curve (AUC) = 0.729, 95% confidence interval (CI) [0.695–0.763]; testing set AUC = 0.722, 95% CI [0.670–0.774]) and calibration (Hosmer-Lemeshow *P* > 0.05). DCA confirmed clinical utility.

**Conclusions:**

This model provides a tool for individualized labor management that aims to reduce unnecessary cesarean deliveries.

## Introduction

Labor pain is a combination of unpleasant sensory, perceptual, and emotional experiences that are associated with autonomic, physiological, emotional, and behavioural responses (Jones et al., 2012). The pain experienced by women varies widely depending upon the sensory and affective perception (Jones et al., 2012; Lam, Leung & Irwin, 2020; Madden et al., 2016).

The psychological responses induced by intense labor pain, such as anxiety and fear, can disrupt the homeostatic equilibrium of both maternal and fetal internal environments, potentially resulting in multiple severe complications for parturients and their offspring. Labor pain may trigger maternal hyperventilation characterized by increased minute ventilation and decreased PaCO_2_ levels, which could precipitate respiratory alkalosis. These pathophysiological alterations may compromise perinatal healthcare quality and even jeopardize maternal-neonatal life safety (Du et al., 2022; Jones et al., 2012; Wong, 2010). Effective analgesia during childbirth is therefore critical for optimizing the maternal delivery experience (Wilson et al., 2018).

Labor pain encompasses both visceral and somatic components. In the early first stage of labor, pain predominantly characterized by visceral nociception arises from distension of the lower uterine segment and cervical dilation. During the late first stage and second stage of labor, the descending fetus applies progressively intensified pressure to the vaginal canal and perineum. This mechanical distension of the birth canal causes intense stretching and tearing of fascial layers and subcutaneous tissues, while simultaneously generating compressive forces on perineal skeletal muscles (Wong, 2010).

Epidural analgesia remains a pivotal modality for pain management during labor worldwide, with neuraxial labor analgesia being recognized as the gold standard demonstrating significant efficacy in pain relief. The World Health Organization (WHO) recommends its use (Callahan et al., 2023; Mazda, 2022; Nanji & Carvalho, 2020). Its use is common: a survey conducted in 13 high-income countries in 2020 found that labor epidural analgesia (LEA) was used in 10% to 83% of labor courses, varying with geography and parity. In the United States, 4 in 5 nulliparous parturients now receive LEA (Seijmonsbergen-Schermers et al., 2020). Notwithstanding its widespread adoption, persisting uncertainties surround optimal epidural anesthesia protocols. During epidural administration, varying drug concentrations and combinations exhibit divergent pharmacodynamic profiles in antepartum and postpartum periods, while their association with intrapartum cesarean delivery (CD) remains controversial (Callahan et al., 2023; Halliday, Nelson & Kearns, 2022; Mazda, 2022). Epidural analgesia has been associated with an elevated risk of CD in clinical studies, while coexisting confounding variables—including maternal characteristics and fetal status—may concurrently potentiate the cesarean risk in parturients receiving this intervention (Herrera-Gómez et al., 2017). Given the multitude of peripartum variables, establishing definitive causality remains methodologically challenging, thus precluding conclusive determination of specific factors directly influencing obstetric decision-making (Anim-Somuah et al., 2018).

Notably, conversion to CD during labor not only heightens maternal and neonatal health risks (Chinese Society of Perinatal Medicine et al., 2025; Sandall et al., 2018), but also contradicts the original intent of epidural analgesia to facilitate vaginal delivery through adequate pain control. Although previous studies have identified factors such as advanced maternal age, overweight/obesity, infertility treatment, multiple pregnancy, and preexisting hypertension as potential contributors to an increased risk of conversion to CD during trial of labor (Panda et al., 2022), research specifically investigating determinants related to epidural analgesia remains limited. This study aims to explore the potential factors associated with CD conversion in parturients receiving epidural analgesia during trial of labor, thereby providing evidence-based guidance for optimizing labor analgesia strategies and reducing unnecessary CD rates.

## Methods

This retrospective cohort study analyzed clinical data of parturients receiving epidural labor analgesia at Zhengzhou Central Hospital Affiliated to Zhengzhou University between January 2022 and December 2023. Data extraction was conducted from January to February 2025, following approval by the Ethics Committee of Zhengzhou Central Hospital Affiliated to Zhengzhou University (Approval No.: ZXYY2024102). The ethics committee confirmed that the study adhered to the Declaration of Helsinki. The trial was registered in the Chinese Clinical Trial Registry (ChiCTR2400091365). Informed consent was waived as the study utilized anonymized data without compromising patient privacy.

### Patient enrollment and grouping

Inclusion criteria: (1) ≥ Age ≥18 years; (2) American Society of Anesthesiologists (ASA) physical status II or III. Exclusion criteria: (1) Gestational age <37 weeks (*i.e.,* non-term pregnancy, as defined by the American College of Obstetricians and Gynecologists); (2) Medically or obstetrically indicated induction of labor; (3) Previous cesarean delivery (excluded due to its dominant confounding effect on cesarean delivery risk Barnea et al., 2025); (4) Pre-analgesia body temperature > 38 °C (as this threshold defines pyrexia (Le Gouez, Benachi & Mercier, 2016); (5) Incomplete medical records; (6) Abnormal analgesic pump data or inconsistent parameter settings. We excluded medically or obstetrically indicated inductions to ensure a homogeneous spontaneously laboring cohort, as induction is itself a significant confounder and independent predictor of CD. Participants were categorized into two groups based on the actual mode of delivery: (1) the Vaginal Delivery Group, which included all patients who achieved a successful vaginal delivery, either spontaneous or instrumental (*e.g.*, vacuum or forceps); and (2) the Cesarean Delivery Group, which comprised all patients who converted to CD during the trial of labor.

### Implementation of epidural labor analgesia

All parturients receiving labor analgesia underwent exclusion of relevant contraindications prior to epidural anesthesia. Risks were comprehensively disclosed, and written informed consent was obtained from the patients or their legal representatives. Epidural catheterization was performed by experienced anesthesiologists at the L2-3 or L3-4 intervertebral spaces. Following successful epidural puncture, a catheter was inserted cephalad for 4–5 cm within the epidural space. A test dose (3–5 mL of 2% lidocaine) was administered through the catheter to confirm the absence of intravascular or subarachnoid placement. Subsequently, the catheter was connected to a patient-controlled analgesia (PCA) pump with an initial bolus infusion. The analgesic solution contained 0.08% ropivacaine combined with 0.4 µg/mL sufentanil, diluted in 0.9% sodium chloride to a total volume of 120 mL. The PCA parameters were configured as follows:

(i) Initial bolus: 10 mL
(ii) Background infusion: eight mL every 50 min
(iii) Patient-controlled bolus dose: eight mL
(iv) Lockout interval: 15 min
(v) Maximum hourly dose: 25 mL

Rescue analgesia was provided by anesthesiologists if patient-activated boluses failed to achieve adequate pain relief (visual analog scale , VAS, ≤3).

### Outcomes

The primary outcome of this study was the rate of conversion to CD during labor. Secondary outcomes included comprehensive observational parameters: (1) Maternal parameters: age, height, weight, pre-pregnancy body mass index, gestational age, parity, and comorbidities (*e.g.*, hypertension, diabetes mellitus); (2) Labor analgesia-related parameters: administration of meperidine prior to epidural analgesia, total dosage of epidural analgesics administered, total PCA button presses and valid PCA demands; and (3) Obstetric parameters: indications for conversion to CD, presence of fetal nuchal cord pre-delivery, use of oxytocin augmentation, cervical dilation at analgesia initiation, estimated fetal weight (assessed by ultrasound at admission prior to analgesia initiation using standardized Hadlock formula calculations Salomon et al., 2019), and incidence of intrapartum fever (defined as tympanic temperature ≥38.0 °C).

### Statistical analysis

Statistical analyses were performed using R software (version 4.2.1;  https://www.r-project.org/). Continuous variables conforming to a normal distribution are presented as mean ± standard deviation (mean ± SD), and comparisons between groups were conducted using the independent samples *t*-test. Continuous variables not conforming to a normal distribution are presented as median (Q1, Q3), and inter-group comparisons were performed using the Wilcoxon rank-sum test. Categorical variables are presented as n (%), and comparisons between groups were made using either Fisher’s exact test or the Chi-square test, as appropriate.

The original dataset was randomly split into training and testing sets at a 7:3 ratio for analytical purposes. Within the training set, 10-fold cross-validation was performed during Least Absolute Shrinkage and Selection Operator (LASSO) regression to enhance model robustness and prevent overfitting. Baseline characteristics were compared between the training and testing sets to confirm no significant differences. Within the training set, univariate analysis was performed between the cesarean delivery group and the vaginal delivery group. Variables demonstrating statistical significance (*P* < 0.05) were included in the LASSO regression analysis. Variables selected by the LASSO analysis were then incorporated into a multivariate logistic regression analysis to construct the prediction model. Prior to multivariate analysis, multicollinearity among candidate predictors was assessed using variance inflation factors (VIF), with VIF > 5 indicating significant collinearity warranting variable exclusion. A nomogram was developed based on this model.

The discriminative ability of the nomogram model was evaluated using the Receiver Operating Characteristic (ROC) curve and quantified by the area under the curve (AUC). Calibration of the nomogram model was assessed using the Hosmer-Lemeshow goodness-of-fit test and calibration curves using the bootstrap method with 1,000 resamples. Decision Curve Analysis (DCA) was employed to evaluate the clinical utility of the model.

## Results

### Participant characteristics

**Figure 1:**
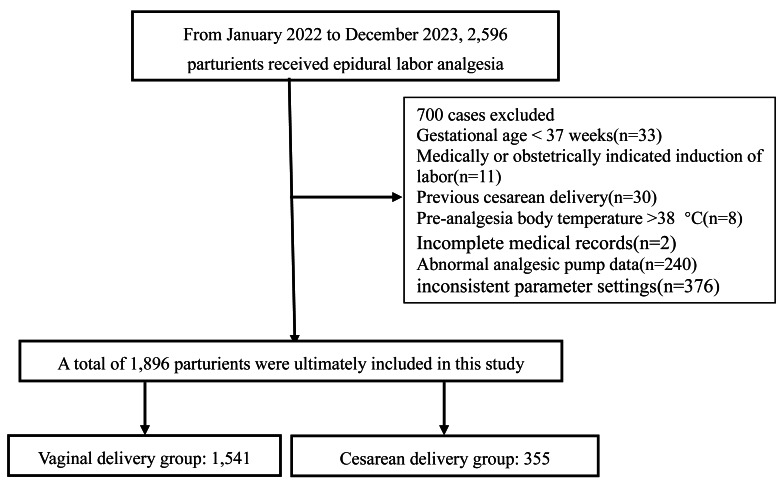
Flow chart illustrating the study population distribution.

A total of 2,596 pregnant women were initially screened. As detailed in Fig. 1, 700 women were excluded based on predefined criteria, resulting in 1896 enrolled participants. Among them, 1,541 women (81.3%) were assigned to the vaginal delivery group, and 355 (18.7%) to the cesarean delivery group. The data were randomly split into training and testing sets at a 7:3 ratio for analytical purposes. Table 1 presents the baseline characteristics stratified by delivery mode (vaginal *vs.* cesarean) and by dataset (training *vs.* testing). The table highlights the significant differences in key predictors between the delivery groups. Univariate analysis confirmed no significant differences in baseline characteristics between the training and testing sets (all *P*-values > 0.05). This ensured comparability for model development and validation.

**Table 1:** (A) Baseline characteristics of the total study population, stratified by delivery mode (vaginal *vs.* cesarean). (B) Comparison of baseline characteristics between the training set and testing set.

(A)				
**Variables**	**Total** **(*n* = 1,896)**	**Vaginal delivery** **(*n* = 1,541)**	**Cesarean delivery** **(*n* = 355)**	***P*** ** value**
**Age, Median (Q1,Q3)**	30 (28, 32)	30 (28, 32)	30 (28, 32)	0.123
**Weight, Median (Q1,Q3)**	70 (65, 76)	70 (64, 76)	71 (66, 77)	0.002^*^
**Height, Median (Q1,Q3)**	163 (160, 166)	163 (160, 166)	162 (159, 165)	0.006^*^
**Pre-pregnancy BMI, Median (Q1,Q3)**	21.3 (19.6, 23.4)	21.2 (19.5, 23.2)	21.8 (20.0, 23.8)	0.003^*^
**Gestational age, n (%)**				<0.001^**^
**<40**	1,069 (56)	903 (59)	166 (47)	
**≥ 40**	827 (44)	638 (41)	189 (53)	
**Primiparity, n (%)**				<0.001^**^
**No**	541 (29)	514 (34)	27 (8)	
**Yes**	1,355 (71)	1,027 (67)	328 (92)	
**Parity, Median (Q1,Q3)**	1 (1, 2)	1 (1, 2)	1 (1, 1)	<0.001^**^
**Hypertension, n (%)**				<0.001^**^
**No**	1,817 (96)	1,490 (97)	327 (92)	
**Yes**	79 (4)	51 (3)	28 (8)	
**Diabetes mellitus, n (%)**				0.175
**No**	1,436 (76)	1,177 (76)	259 (73)	
**Yes**	460 (24)	364 (24)	96 (27)	
**Fetal nuchal cord, Median (Q1,Q3)**	0 (0, 1)	0 (0,1)	1 (1,1)	0.324
**Meperidine use, n (%)**				<0.001^**^
**No**	1,795 (95)	1,474 (96)	321 (90)	
**Yes**	101 (5)	67 (4)	34 (10)	
**Oxytocin augmentation, n (%)**				0.002^*^
**No**	604 (32)	516 (33.5)	88 (25)	
**Yes**	1,292 (68)	1,025 (66.5)	267 (75)	
**Cervical dilation at analgesia initiation, Median (Q1,Q3)**	2 (2, 3)	2 (2, 3)	2 (2, 2)	<0.001^**^
**Intrapartum fever, n (%)**				<0.001^**^
**No**	1,734 (91)	1,448 (94)	286 (81)	
**Yes**	162 (9)	93 (6)	69 (19)	
**Estimated fetal weight, Median (Q1,Q3)**	3,317.5 (3,090, 3,565)	3,290 (3,070, 3,540)	3,450 (3,190, 3,680)	<0.001^**^
**Valid PCA demands, Median (Q1,Q3)**	0 (0, 1)	0 (0, 1)	0 (0, 1)	0.052
**Total PCA demands, Median (Q1,Q3)**	0 (0, 2)	0 (0, 2)	0 (0, 1)	0.004^*^
**Total epidural analgesic consumption, Median (Q1,Q3)**	56 (32, 88)	56 (32, 88)	60 (40, 88)	0.007^*^

(B)			
**Variables**	**Training set (*n* = 1,328)**	**Test set (*n* = 568)** **(*n* = 568)**	***P*** ** value**
**Age, Median (Q1,Q3)**	30 (28, 32)	30 (28, 32)	0.577
**Weight, Median (Q1,Q3)**	70 (65, 76)	70 (65, 76)	0.382
**Height, Median (Q1,Q3)**	163 (160, 166)	162.5 (160, 165)	0.659
**Pre-pregnancy BMI, Median (Q1,Q3)**	21.27 (19.6, 23.3)	21.38 (19.53, 23.42)	0.642
**Gestational age, n (%)**			0.82
**<40**	746 (56)	323 (57)	
**≥ 40**	582 (44)	245 (43)	
**Primiparity, n (%)**			0.41
**No**	371 (28)	170 (30)	
**Yes**	957 (72)	398 (70)	
**Parity, Median (Q1,Q3)**	1 (1, 2)	1 (1, 2)	0.305
**Hypertension, n (%)**			0.967
**No**	1,272 (96)	545 (96)	
**Yes**	56 (4)	23 (4)	
**Diabetes mellitus, n (%)**			0.843
**No**	1,008 (76)	428 (75)	
**Yes**	320 (24)	140 (25)	
**Fetal nuchal cord, Median (Q1,Q3)**	0 (0, 1)	0 (0, 1)	0.78
**Meperidine use, n (%)**			0.538
**No**	1,254 (94)	541 (95)	
**Yes**	74 (6)	27 (5)	
**Oxytocin augmentation, n (%)**			0.867
**No**	421 (32)	183 (32)	
**Yes**	907 (68)	385 (68)	
**Cervical dilation at analgesia initiation, Median (Q1,Q3)**	2 (2, 3)	2 (2, 3)	0.885
**Intrapartum fever, n (%)**			0.211
**No**	1,222 (92)	512 (90)	
**Yes**	106 (8)	56 (10)	
**Estimated fetal weight, Median (Q1,Q3)**	3,317.5 (3,098.75, 3,565)	3,317.5 (3,080, 3,561.25)	0.664
**Valid PCA demands, Median (Q1,Q3)**	0 (0, 1)	0 (0, 1)	0.262
**Total PCA demands, Median (Q1,Q3)**	0 (0, 2)	1 (0, 2)	0.281
**Total epidural analgesic consumption, Median (Q1,Q3)**	56 (32, 88)	56 (32, 88)	0.552

**Notes.**

Continuous variables are expressed as medians (interquartile range) and categorical variables are presented as frequencies (%).

* *p* < 0.05.

** *p* < 0.001, indicate statistical significance.

### Univariate analysis of factors associated with CD in the training set

Univariate analysis within the training set revealed significant differences (*P* < 0.05) between the cesarean delivery and vaginal delivery groups for multiple variables (see Table S1). Key findings indicated that, compared to the vaginal delivery group, the cesarean delivery group had lower maternal height, higher pre-pregnancy BMI, a higher proportion of gestational age ≥40 weeks, a higher rate of primiparas, hypertension, meperidine use, intrapartum fever, and a smaller cervical dilation at analgesia initiation, as well as higher estimated fetal weight. Additionally, significant differences were observed in oxytocin augmentation usage, total PCA bolus demands, and total dosage of epidural analgesics administered between groups. All details including medians, interquartile range (IQRs), percentages, and exact *P*-values are provided in Table S1.

### Prediction model development

**Figure 2:**
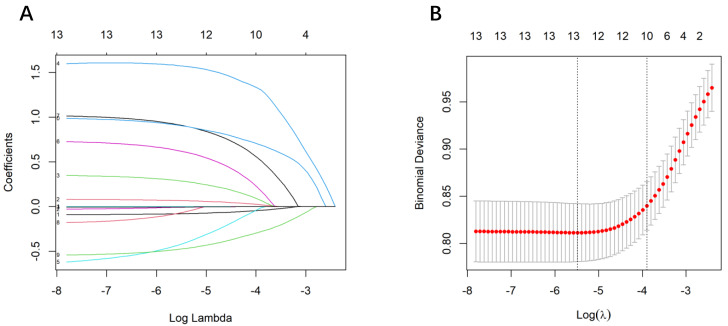
Variable selection by LASSO binary logistic regression model. A coefficient profile plot was produced against the log(lambda) sequence (A). Eight variables with nonzero coefficients were selected by optimal lambda. By verifying the optimal parameter (lambda) in the LASSO model, the partial likelihood deviance (binomial deviance) curve was plotted *versus* log(lambda) and dotted vertical lines were drawn based on the 1 standard error criterion (B).

The 14 variables with significant associations in the training set were included in a LASSO regression analysis. Based on the training set data (see Figs. 2A and 2B), the LASSO algorithm identified 10 predictors with nonzero coefficients. These predictors were: height, pre-pregnancy BMI, gestational age ≥40 weeks, primiparity, parity, hypertension, meperidine use, cervical dilation at analgesia initiation, intrapartum fever, and estimated fetal weight.

Subsequent multivariate logistic regression analysis of these 10 variables identified the following eight as independent predictors of the outcome: height, pre-pregnancy BMI, gestational age ≥ 40 weeks, hypertension, meperidine use, cervical dilation at analgesia initiation, intrapartum fever, and estimated fetal weight (all *p***-**values < 0.05; see Table 2).

**Figure 3:**
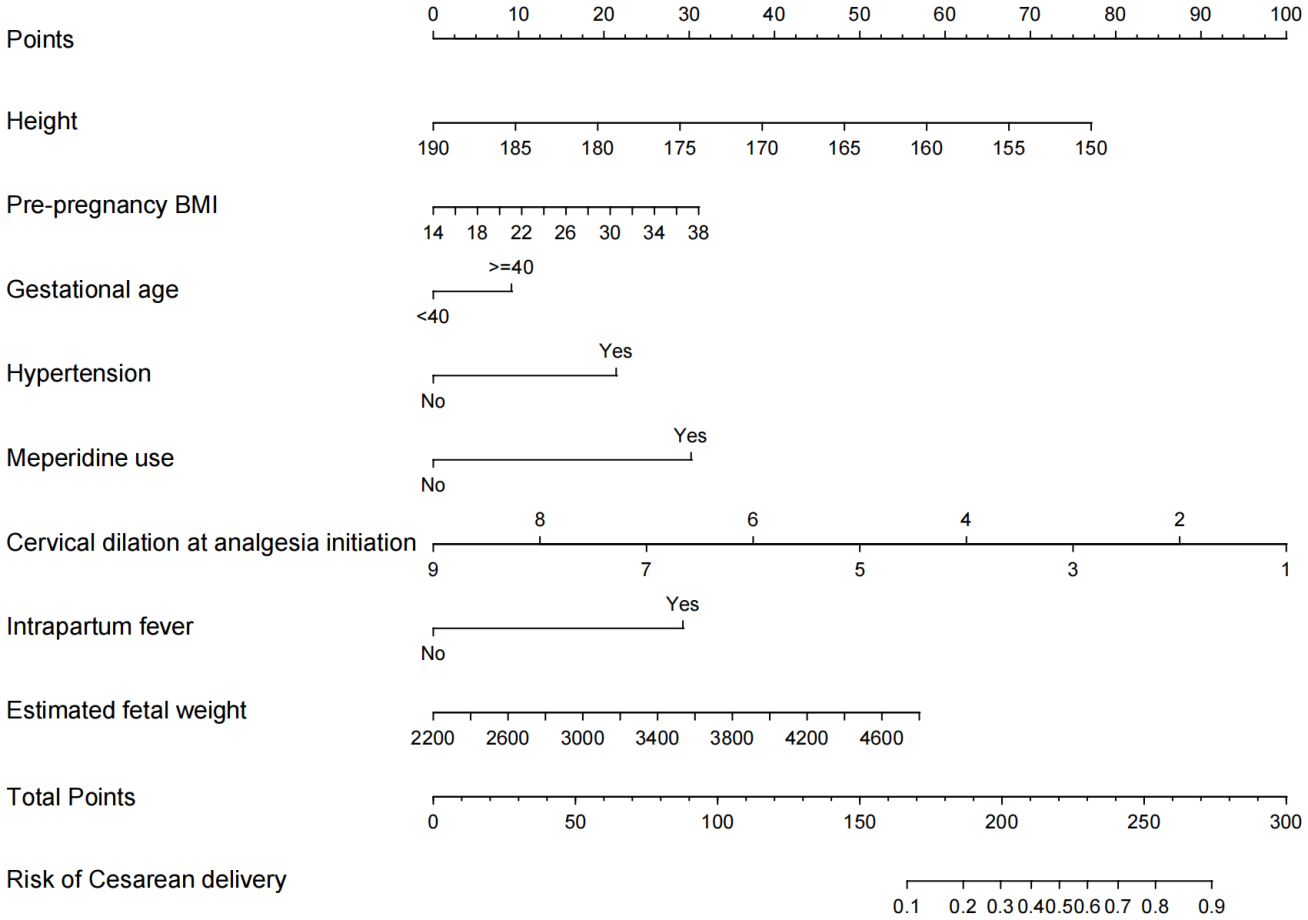
Development of the risk nomogram. Predictors in the nomogram for intrapartum cesarean delivery risk among women receiving LEA include: height, pre-pregnancy BMI, gestational age ≥40 weeks, hypertension, meperidine use, cervical dilation at analgesia initiation, Intrapartum fever, estimated fetal weight.

The multivariate logistic regression model, which incorporated these eight significant variables, was used as the final prediction model. A nomogram was developed to visualize the final model (Fig. 3).

### Prediction model validation

**Figure 4:**
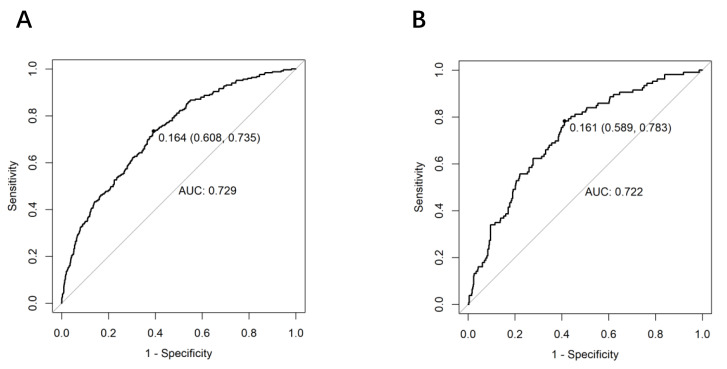
ROC curve validation of the predictive model for intrapartum cesarean delivery risk in women receiving LEA, based on the nomogram. The *y*-axis meant the true-positive rate of the risk prediction. The *x*-axis meant the false-positive rate of the risk prediction. The black line represented the performance of the nomogram. (A) From the training set and (B) from the validation set.

The discriminative ability of the nomogram was evaluated using receiver operating characteristic (ROC) curves. In the training set, AUC was 0.729 (95% CI [0.695–0.763]), with a sensitivity of 73.5% and a specificity of 60.8% at the optimal cutoff value of 0.164 (Fig. 4A). The testing set yielded an AUC of 0.722 (95% CI [0.670–0.774]), with a sensitivity of 78.3% and a specificity of 58.9% at the optimal cutoff value of 0.161 (Fig. 4B).

**Figure 5:**
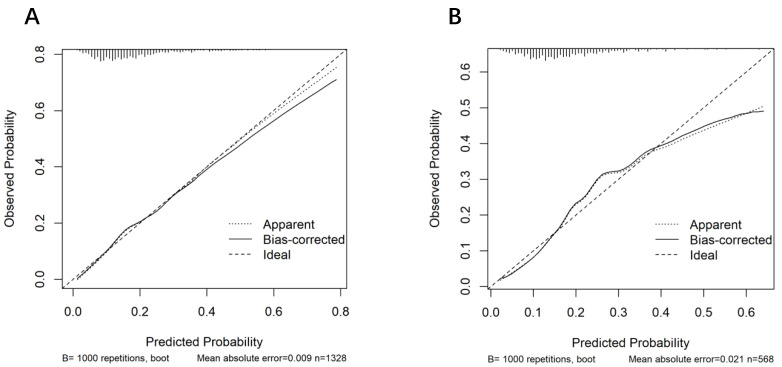
Calibration curves of the nomogram for predicting intrapartum cesarean delivery risk in women receiving LEA. The *x*-axis represents the predicted risk of intrapartum cesarean delivery (range 0–1). The *y*-axis represents the observed probability of intrapartum cesarean delivery (range 0–1). The diagonal dotted line indicates a perfect prediction by an ideal model. The solid line in subfigure (A) represents the performance of the training set, and in subfigure (B), the performance of the validation set; a closer fit of the solid line to the diagonal dotted line indicates better prediction accuracy. Bootstrap sampling with 1,000 replicates was performed, yielding a mean absolute error of 0.021, and the sample size was *n* = 568.

Calibration was assessed using the Hosmer-Lemeshow goodness-of-fit test and calibration curves (Bootstrap method with 1,000 resamples). The results showed good calibration in both sets (training set: *χ*^2^ = 8.02, *P* = 0.43; testing set: *χ*^2^ = 10.40, *P* = 0.24; both *P* > 0.05) (Fig. 5).

**Figure 6:**
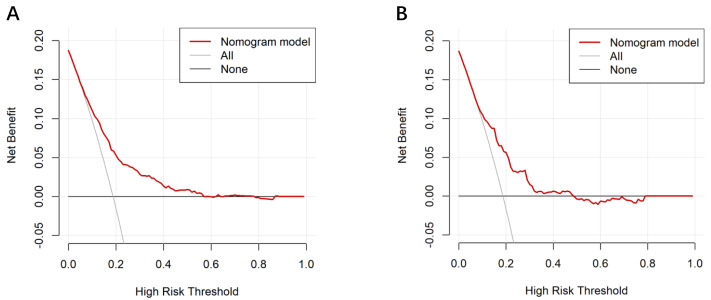
Decision curve analysis of the nomogram for predicting intrapartum cesarean delivery risk in women receiving LEA. The *y*-axis measured the net benefit. The thick solid line represented the assumption that all patients had no intrapartum cesarean delivery. The thin solid line represented the assumption that all patients had intrapartum cesarean delivery. The red line represented the risk nomogram. (A) From the training set and (B) from the validation set.

DCA demonstrated that the nomogram provided a positive net benefit across a wide range of threshold probabilities (0–56% in the training set and 0–48% in the testing set), demonstrating its clinical utility in guiding interventions (*e.g.*, intensified monitoring or preemptive preparation for CD) compared with the treat-all and treat-none strategies (Fig. 6). For practical implementation, this nomogram can be deployed as a bedside calculator or integrated into a mobile application to facilitate real-time risk stratification during labor admission assessment, thereby supporting personalized clinical decision-making.

## Discussion

In this study, 18.7% of parturients women receiving epidural labor analgesia underwent intrapartum conversion to cesarean delivery (CD). Multivariate analysis identified the following variables as significantly associated with conversion: height, pre-pregnancy BMI, gestational age ≥ 40 weeks, hypertension, meperidine use, cervical dilation at analgesia initiation, intrapartum fever, and estimated fetal weight. Using these eight clinically available predictors, we constructed and validated a practical prediction tool for intrapartum cesarean conversion in women with epidural analgesia. The model demonstrated good performance in terms of discrimination and calibration, highlighting its potential clinical utility when implemented as a nomogram.

### Independent risk factor analysis

Higher pre-pregnancy BMI was significantly associated with increased incidence of macrosomia, postpartum hemorrhage, labor complications, CD, and gestational diabetes mellitus (GDM). Strict weight management is recommended for these women to reduce obesity-related obstetric and neonatal risks (Zhang et al., 2023).

**Table 2:** Multivariable analysis of factors associated with intrapartum cesarean delivery (training set).

**Characteristic**	**OR** ^1^	**95% CI** ^1^	***p*** **-value**
**Height**	0.91	0.88, 0.95	<0.001
**Pre-pregnancy BMI**	1.08	1.03, 1.14	0.003
**Gestational age**			0.027
***<40***	–	–	
***>=40***	1.42	1.04, 1.93	
**Primiparity**			0.3
***No***	–	–	
***Yes***	4.46	0.22, 30.7	
**Parity**	0.55	0.03, 2.81	0.5
**Hypertension**			0.030
***No***	–	–	
***Yes***	2.10	1.08, 3.99	
**Meperidine use**			<0.001
***No***	–	–	
***Yes***	2.84	1.65, 4.86	
**Cervical dilation at analgesia initiation**	0.61	0.50, 0.74	<0.001
**Intrapartum fever**			<0.001
***No***	–	–	
***Yes***	2.51	1.61, 3.92	
**Estimated fetal weight**	1.00	1.00, 1.00	<0.001

**Notes.**

1 OR, Odds Ratio; CI, Confidence Interval.

Although obesity is not a direct indication for CD, it strongly correlates with prolonged labor, increased oxytocin requirements, and higher cesarean rates (Tan & Habib, 2021). Obese women are twice as likely to undergo CD, potentially due to slower cervical dilation, higher comorbidity rates, increased risk of shoulder dystocia, and excessive gestational weight gain (Creanga, Catalano & Bateman, 2022). Additionally, gestational age ≥40 weeks and fetal weight gain are associated with higher cesarean rates, with the former likely influencing birth weight (Herrera-Gómez et al., 2017). Elevated pre-pregnancy BMI is a major independent risk factor for fetal macrosomia (birth weight >4,000 g) (Creanga, Catalano & Bateman, 2022; Zhang et al., 2023). Macrosomia can lead to labor dystocia (including shoulder dystocia) through biomechanical mechanisms such as cephalopelvic disproportion. The risk of shoulder dystocia is 2 to 2.5 times higher in women with a BMI ≥35 (Creanga, Catalano & Bateman, 2022). This pathophysiological pathway contributes to increased oxytocin requirements and a significantly elevated risk of CD (approximately double the risk in obese compared to normal-weight women), often in conjunction with other common comorbidities (Creanga, Catalano & Bateman, 2022).

Primiparity, due to lack of childbirth experience, may lead to maternal anxiety, poor cooperation during labor, physical exhaustion, and subsequent uterine inertia or fetal distress, ultimately increasing the likelihood of CD (Ghafari-Saravi et al., 2022). Although both primiparity and parity were initially selected by LASSO regression as potential predictors, neither retained statistical significance in the final multivariate logistic model due to significant multicollinearity (VIF > 9.5 for both). In contrast, cervical dilation at analgesia initiation showed low collinearity (VIF = 1.016) and served as a more objective and individualized measure of labor progress, thereby superseding the predictive contribution of parity status in the model.

Clinically, although primiparity is a known risk factor for prolonged labor, cervical dilation at analgesia initiation quantifies actual progression at a critical decision point. For example, a primiparous woman with adequate cervical dilation (*e.g.*, ≥4 cm) may have a lower conversion risk than one with limited dilation (<3 cm), explaining why this dynamic indicator outperforms static parity status in our model.

Pregnancy complications such as cardiovascular disease, diabetes, and hypertension increase labor complexity. Hypertensive disorders are linked to higher cesarean rates, preterm birth, low birth weight, and fetal mortality (Ramos Filho & Antunes, 2020). GDM is associated with increased risks of CD, preterm birth, low Apgar scores, macrosomia, and large-for-gestational-age infants (Ye et al., 2022). In this study, the proportion of GDM was higher in the cesarean group (27.0% *vs.* 23.6%), although this difference was not statistically significant, possibly due to insufficient sample size.

Opioids such as meperidine, although widely used for labor analgesia, can cause adverse effects including maternal nausea/vomiting, neonatal respiratory depression, and breastfeeding difficulties (Zuarez-Easton et al., 2023). The ACOG guidelines explicitly discourage meperidine for labor analgesia due to potential neonatal toxicity from its metabolites (American College of Obstetricians and Gynecologists, 2019). In this cohort, the cesarean group had a higher proportion of meperidine use prior to analgesia (9.6% *vs.* 4.3%).

Intrapartum fever emerged as a critical risk factor for CD in women receiving epidural analgesia. The cesarean group exhibited a significantly higher rate of fever (19.4% *vs.* 6.0%). Intrapartum fever is associated with increased infectious morbidity, often leads to the empirical use of antibiotics due to diagnostic uncertainty, and contributes to uterine atony, thereby elevating the risks of both CD and postpartum hemorrhage by 2- to 3-fold (Goetzl, 2023). Fever ≥38.0 °C triggers a systemic inflammatory response, which can disrupt normal uterine contractility. This pathophysiological cascade significantly increases the risk for CD. Once intrapartum fever is diagnosed, prompt antibiotic therapy is recommended due to the inability to reliably exclude an infectious etiology. However, prophylactic acetaminophen is ineffective in preventing its occurrence.

### Analysis of protective factors

The risk of CD decreased with increasing maternal height. Height is positively correlated with pelvic dimensions, as women with shorter stature are at higher risk of cephalopelvic disproportion due to anatomical constraints, which can potentially lead to obstructed labor from fetal head or shoulder impaction (Marbaniang, Lhungdim & Chaurasia, 2022). Earlier initiation of epidural analgesia was associated with higher cesarean rates in some studies, while others found no association with the timing of analgesia (Chestnut et al., 1994; Lieberman et al., 1996; Malone et al., 1996; Ohel et al., 2006; Olofsson et al., 1998). In our multivariate analysis, greater cervical dilation at analgesia initiation was associated with a reduced risk of intrapartum CD. Despite inconsistent conclusions regarding optimal analgesic timing, the ASA Practice Guidelines for Obstetric Anesthesia recommend early analgesic provision upon maternal request (American College of Obstetricians and Gynecologists, 2019). In our study cohort, this ASA recommendation was implemented as a standard protocol. Epidural analgesia was initiated solely upon patient request, and the institution did not enforce a policy of delaying administration based on cervical dilation or other obstetric parameters.

## Limitations

This study has several limitations. First, as a single-center retrospective study conducted in a regional population (Henan Province, China), it may be subject to selection bias and unmeasured confounders specific to obstetric anesthesia practice (*e.g.*, labor support quality, patient pain tolerance thresholds, clinician decision-making patterns). Second, the model did not incorporate dynamic labor progression parameters (*e.g.*, cervical dilation rate) and lacks external validation, further limiting its generalizability across diverse populations. Third, the model demonstrated an AUC of approximately 0.73, which is considered acceptable but indicates room for improvement. Additionally, certain indicators (*e.g.*, PCA bolus frequency and total analgesic consumption) did not reach statistical significance. These variables were included based on the hypothesis that frequent PCA demands may indicate inadequate analgesia and breakthrough pain, which could contribute to CD conversion; however, this association was not confirmed in our model. These findings suggest the need for larger sample sizes and additional predictors to enhance predictive accuracy. Future refinements should integrate dynamic prediction modeling (*i.e.,* real-time risk score updating based on evolving labor parameters) and cost-effectiveness analyses (*e.g.*, comparing resource utilization between model-guided interventions *vs.* standard care).

Despite these limitations, the wide range of threshold probabilities over which the model demonstrated clinical utility, as shown by the DCA, provides a rationale for implementing our model as a clinical tool. We propose that the nomogram developed in this study could be operationalized as an online calculator or a mobile application. This would allow anesthesiologists to rapidly estimate an individual patient’s risk of CD during intrapartum assessment, thereby facilitating clinical decision-making. Prior to widespread clinical implementation, the essential next step is external validation in a more diverse population.

## Conclusion

We developed and validated a predictive model for intrapartum conversion to cesarean delivery in women receiving epidural labor analgesia, incorporating the following eight variables: maternal height, pre-pregnancy BMI, gestational age ≥ 40 weeks, hypertension, meperidine use, cervical dilation at analgesia initiation, intrapartum fever, and estimated fetal weight. This model provides a practical tool for anesthesiologists and obstetricians to identify women at high risk for intrapartum cesarean conversion among women planning epidural analgesia. Based on the individualized risk assessment, clinicians can optimize resource allocation and consider preemptive measures for high-risk parturients. Future efforts should focus on external validation in multicenter and diverse populations and incorporation of dynamic intrapartum variables to further enhance the model’s clinical utility and generalizability.

##  Supplemental Information

10.7717/peerj.20358/supp-1 Supplemental Information 1 Maternal data of the vaginal delivery group and cesarean section group

10.7717/peerj.20358/supp-2 Supplemental Information 2 Univariate analysis comparing the cesarean delivery group and the vaginal delivery group within the training set

10.7717/peerj.20358/supp-3 Supplemental Information 3 STROBE checklist

## Additional Information and Declarations

### Data Availability

The following information was supplied regarding data availability: The raw data are available in the Supplemental File.
