# Supplementary material for: Risk factors and predictive model for intrapartum cesarean delivery in women with epidural analgesia: a retrospective cohort study
Source: PeerJ. 2025 Nov 19;13:e20358. doi: 10.7717/peerj.20358 (PMC12640134; doi:10.7717/peerj.20358)
Supplement: Supplemental Information 2 [file peerj-13-20358-s002.docx]

| Variables | Total (n = 1328) | Cesarean delivery (n = 249) | Vaginal delivery (n = 1079) | p | statistic |
| --- | --- | --- | --- | --- | --- |
| Age, Median (Q1,Q3) | 30 (28, 32) | 30 (28, 32) | 30 (28, 32) | 0.581 | 131336 |
| Weight, Median (Q1,Q3) | 70 (65, 76) | 71 (65, 76.5) | 70 (64, 75.5) | 0.05 | 145003 |
| Height, Median (Q1,Q3) | 163 (160, 166) | 161 (158, 165) | 163 (160, 166) | **< 0.001** | 110211.5 |
| Pre-pregnancy BMI, Median (Q1,Q3) | 21.27 (19.6, 23.3) | 21.75 (20.1, 23.63) | 21.2 (19.53, 23.2) | **0.008** | 148767.5 |
| Gestational age, n (%) |  |  |  | **0.002** | 10.052 |
| <40 | 746 (56) | 117 (47) | 629 (58) |  |  |
| >=40 | 582 (44) | 132 (53) | 450 (42) |  |  |
| Primiparity, n (%) |  |  |  | **< 0.001** | 69.129 |
| No | 371 (28) | 16 (6) | 355 (33) |  |  |
| Yes | 957 (72) | 233 (94) | 724 (67) |  |  |
| Parity, Median (Q1,Q3) | 1 (1, 2) | 1 (1, 1) | 1 (1, 2) | **< 0.001** | 98707 |
| Hypertension, n (%) |  |  |  | **0.014** | 5.996 |
| No | 1272 (96) | 231 (93) | 1041 (96) |  |  |
| Yes | 56 (4) | 18 (7) | 38 (4) |  |  |
| Diabetes mellitus, n (%) |  |  |  | 0.565 | 0.331 |
| No | 1008 (76) | 185 (74) | 823 (76) |  |  |
| Yes | 320 (24) | 64 (26) | 256 (24) |  |  |
| Fetal nuchal cord, Median (Q1,Q3) | 0 (0, 1) | 0 (0, 1) | 0 (0, 1) | 0.178 | 140331 |
| Meperidine use, n (%) |  |  |  | **< 0.001** | 20.093 |
| No | 1254 (94) | 220 (88) | 1034 (96) |  |  |
| Yes | 74 (6) | 29 (12) | 45 (4) |  |  |
| Oxytocin augmentation, n (%) |  |  |  | **0.042** | 4.122 |
| No | 421 (32) | 65 (26) | 356 (33) |  |  |
| Yes | 907 (68) | 184 (74) | 723 (67) |  |  |
| Cervial dilation at analgesia initiation, Median (Q1,Q3) | 2 (2, 3) | 2 (2, 2) | 2 (2, 3) | **< 0.001** | 102478.5 |
| Intrapartum fever, n (%) |  |  |  | **< 0.001** | 47.706 |
| No | 1222 (92) | 202 (81) | 1020 (95) |  |  |
| Yes | 106 (8) | 47 (19) | 59 (5) |  |  |
| Estimated fetal weight, Mean ± SD | 3328.53 ± 355.13 | 3414.81 ± 349.75 | 3308.61 ± 353.55 | **< 0.001** | 4.31 |
| Valid PCA demands, Median (Q1,Q3) | 0 (0, 1) | 0 (0, 1) | 0 (0, 1) | 0.184 | 127905.5 |
| Total PCA demands, Median (Q1,Q3) | 0 (0, 2) | 0 (0, 1) | 0 (0, 2) | **0.032** | 123564.5 |
| Total epidural analgesic consumption, Median (Q1,Q3) | 56 (32, 88) | 62 (42, 88) | 55 (32, 88) | **0.004** | 149859 |
